# Metabotropic Glutamate Receptor-1 Contributes to Progression in Triple Negative Breast Cancer

**DOI:** 10.1371/journal.pone.0081126

**Published:** 2014-01-03

**Authors:** Malathi Banda, Cecilia L. Speyer, Sara N. Semma, Kingsley O. Osuala, Nicole Kounalakis, Keila E. Torres Torres, Nicola J. Barnard, Hyunjin J. Kim, Bonnie F. Sloane, Fred R. Miller, James S. Goydos, David H. Gorski

**Affiliations:** 1 Department of Surgery, Wayne State University School of Medicine, Detroit, Michigan, United States of America; 2 Barbara Ann Karmanos Cancer Institute, Detroit, Michigan, United States of America; 3 Department of Pharmacology, Wayne State University School of Medicine, Detroit, Michigan, United States of America; 4 Division of Surgical Oncology, UMDNJ-Robert Wood Johnson University Medical School, New Brunswick, New Jersey, United States of America; 5 Department of Pathology, UMDNJ-Robert Wood Johnson University Medical School, New Brunswick, New Jersey, United States of America; 6 The Cancer Institute of New Jersey, New Brunswick, New Jersey, United States of America; 7 Department of Oncology, Wayne State University School of Medicine, Detroit, Michigan, United States of America; University of South Alabama, United States of America

## Abstract

TNBC is an aggressive breast cancer subtype that does not express hormone receptors (estrogen and progesterone receptors, ER and PR) or amplified human epidermal growth factor receptor type 2 (HER2), and there currently exist no targeted therapies effective against it. Consequently, finding new molecular targets in triple negative breast cancer (TNBC) is critical to improving patient outcomes. Previously, we have detected the expression of metabotropic glutamate receptor-1 (gene: *GRM1*; protein: mGluR1) in TNBC and observed that targeting glutamatergic signaling inhibits TNBC growth both *in vitro* and *in vivo*. In this study, we explored how mGluR1 contributes to TNBC progression, using the isogenic MCF10 progression series, which models breast carcinogenesis from nontransformed epithelium to malignant basal-like breast cancer. We observed that mGluR1 is expressed in human breast cancer and that in MCF10A cells, which model nontransformed mammary epithelium, but not in MCF10AT1 cells, which model atypical ductal hyperplasia, mGluR1 overexpression results in increased proliferation, anchorage-independent growth, and invasiveness. In contrast, mGluR1 knockdown results in a decrease in these activities in malignant MCF10CA1d cells. Similarly, pharmacologic inhibition of glutamatergic signaling in MCF10CA1d cells results in a decrease in proliferation and anchorage-independent growth. Finally, transduction of MCF10AT1 cells, which express c-Ha-*ras*, using a lentiviral construct expressing *GRM1* results in transformation to carcinoma in 90% of resultant xenografts. We conclude that mGluR1 cooperates with other factors in hyperplastic mammary epithelium to contribute to TNBC progression and therefore propose that glutamatergic signaling represents a promising new molecular target for TNBC therapy.

## Introduction

Despite advances in diagnosis and treatment over the last three decades, breast cancer remains the second most common cause of cancer death in women [1]. Approximately 15–20% of breast cancers do not express hormone receptors (estrogen or progesterone receptors, ER or PR) or amplify human epidermal growth factor receptor 2 (HER2). Although it is heterogeneous [2], this form of breast cancer, dubbed “triple negative breast cancer” (TNBC), shows considerable overlap with the “basal-like” subtype [3]–[5] and tends to be the most aggressive form with the poorest prognosis [6]. Because it is more likely to metastasize early and recur rapidly after treatment [7], TNBC results in a disproportionate number of breast cancer deaths [6]. The therapeutic challenge to clinicians is that TNBC does not respond to drugs targeting hormone receptors or HER2, leaving cytotoxic chemotherapy as the only current option for systemic treatment [8]. That is why finding new molecular targets will be essential to improving outcomes in women with TNBC. In this study, we build on our initial report [9] that glutamatergic signaling represents a potential therapeutic target in TNBC in a preclinical model of TNBC progression by presenting evidence suggesting that mGluR1 can strongly contribute to malignant behavior and progression in nontransformed mammary epithelium.

Metabotropic glutamate receptors (genes: ***GRM1*** **-** ***GRM8***; receptors: **mGluR1-mGluR8**) belong to a family of G-protein-coupled seven transmembrane domain receptors (GPCRs) [10] that mediate responses to a diverse array of signaling molecules, including hormones, neurotransmitters, chemokines, and autocrine and paracrine factors [11]–[14]. Pollock *et al* [15] first reported a potential mechanistic link between glutamatergic signaling and cancer after observing in a transgenic mouse model that dysregulated mGluR1 activity results in melanoma formation with high penetrance [15]–[17]. Consistent with these observations, pharmacological inhibition of glutamatergic signaling blocks the growth of melanoma cell lines and xenografts [16], [18]–[20], and mGluRs have been implicated as novel drivers of oncogenesis in melanoma and other tumor types, with somatic mutations that alter downstream mGluR1 intracellular localization signaling having been recently reported [21], [22].

Our earlier results suggested that glutamatergic signaling is a potential therapeutic target in TNBC. For example, we reported that inhibitors of glutamatergic signaling block TNBC cell proliferation in a time- and dose-dependent manner correlating with increased apoptosis *in vitro* [9]. These same compounds are also effective against MDA-MB-231 xenografts in mice, which express mGluR1 [9]. One of these inhibitors, riluzole, is a glutamate release inhibitor in current clinical use that is FDA-approved for amyotropic lateral sclerosis [23]. To explore how mGluR1 might contribute to progression in TNBC and how better we might target it, we evaluated mGluR1 function in a progression series of isogenic triple negative cell lines derived from MCF10A cells. The parental cell line (MCF10A) having been originally isolated from a woman with fibrocystic change [24], the members of the MCF10A series resemble the basal-like subtype [25]–[27], express neither ER, PR, nor HER2, and recapitulate the stages of breast carcinogenesis [28], making these cell lines a reasonable *in vitro* model for TNBC progression [29], [30]. In our experiments, blocking glutamatergic signaling inhibited the growth of the malignant members of the progression series, and mGluR1 expression contributed to malignant behavior in one of the benign members of the series. Based on these data, we conclude that mGluR1 can interact with other factors to promote progression in hyperplastic mammary epithelium and therefore represents a potentially promising therapeutic target in TNBC.

## Materials and Methods

### Ethics statement

All animal experiments were approved by the Wayne State University Institutional Animal Care and Use Committee (IACUC) and are encompassed by approved animal protocol #A03-03-11. Veterinary care is provided 24 hours a day by one of several vivariums located at Wayne State University. Vivariums are regularly inspected by the U.S. Department of Agriculture (USDA) Animal and Plant Health Inspection Service Animal Care, and are accredited by the Association for Assessment and Accreditation of Laboratory Animal Care International. The animals were subjected to no unnecessary discomfort, pain, or injury during these studies. In order to minimize the possibility of pain and suffering, mice were euthanized when tumors reached a volume of 1,600 mm^3^. Anesthesia and/or tranquilizing agents (intraperitoneal ketamine and xylene) were used when indicated and appropriate to minimize pain and discomfort. Harvesting of tissue was performed only after the animals had been euthanized by CO_2_ inhalation and cervical dislocation. Tissue microarrays from breast cancer tissue were constructed under an exempt protocol, specifically Exemption #4 (research involving the collection or study of existing data, documents, records, pathological specimens, or diagnostic specimens, if these sources are publicly available or if the information is recorded by the investigator in such a manner that subjects cannot be identified, directly or through identifiers linked to the subjects) confirmed by the UMNDJ-Robert Wood Johnson Medical School Institutional Review Board. The tissue specimens used were pre-existing samples that had been de-identified.

### Cell Culture and reagents

The MCF10A series of cell lines were originally developed at the Michigan Cancer Foundation (the institutional precursor to the Karmanos Cancer Institute) and are available to us from original stocks at low passage [28], [31]–[33]. MCF10A series cell lines were cultured in DMEM/F12 (1∶1) media (Life Technologies, Carlsbad, CA) supplemented with 5% horse serum, 10 µg/ml insulin, 20 µg/ml EGF, 0.5 µg/ml hydrocortisone and 100 ng/ml cholera toxin plus antibiotics at 37°C, 5% CO_2_. MCF10A and MCF10AT1 transduced with pLenti-*GRM1* or control *LacZ* were grown with 5 to 8 µg/ml blasticidin (Life Technologies, Carlsbad, CA); MCF10DCIS.com and MCF10CA1d transduced with lentiviral GIPZ sh*GRM1* or non-silencing control were maintained in 2 µg/ml puromycin (Life Technologies, Carlsbad, CA). The noncompetitive mGluR1 inhibitor BAY36-7620 [(3aS,6aS)-6a-naphtalen-2-ylmethyl-5-methyliden-hexahydro-cyclopental [c]-furan-1-on], competitive mGluR1 inhibitor LY367385 [(*S*)-(+)-α-Amino-4-carboxy-2-methylbenzeneacetic acid], and mGluR1 agonist L-quisqualate [(L)-(+)-a-amino-3,5-dioxo-1,2,4-oxadiazolidine-2-propanoic acid] were purchased from Tocris Bioscience (Ellisville, MO, USA).

### Constructs

*GRM1* inserted in PCI-Neo vector was a kind gift from Suzie Chen (Rutgers, The State University of New Jersey, Piscataway). *GRM1* was inserted into the pLenti6.3/V5-TOPO® cloning vector (Life Technologies, Carlsbad, CA) to produce pLenti-*GRM1*, and pLenti-LacZ was used as a control. To silence *GRM1* expression, we have previously validated five shRNA constructs targeting human *GRM1* from the Thermo Scientific Open Biosystems Human GIPZ Lentiviral shRNAmir library (Lafayette, CO) for their ability to knock down *GRM1* expression prior to use [9]. Approximately 5×10^5^ TU/ml of either pLenti6.3/V5-TOPO® *GRM1* or *LacZ* control were used to infect selected cell lines, which then underwent selection using blasticidin, with measurements performed on pooled samples of transduced cells. For overexpression, cells were used approximately 3 to 8 passages after infection with expression vector. For silencing, cells were used 2 to 4 passages after infection. *GRM1* and mGluR1 expression were confirmed by QRT-PCR and Western blot, respectively according to previously published methods [9]. To silence *GRM1*, the Trans-Lentiviral GIPZ Packaging System (Thermo Scientific Open Biosystems, Lafayette, CO) was used to generate viral particles according to the manufacturer's protocol. Approximately 5×10^5^ TU/ml of sh*GRM1* was used to infect cells, which then underwent short term selection with puromycin, after which pooled cell populations were used as with the overexpression experiments. Non-silencing vector was used as a control. Knockdown was verified by qRT-PCR and Western blot.

### Human tumor samples

We obtained 49 breast cancer and 10 normal breast specimens from reduction mammoplasties under an IRB-approved protocol from the Tissue Retrieval Service (TRS), a shared resource of The Cancer Institute of New Jersey. Tissue specimens were coded to protect patient confidentiality according to HIPAA regulations. The only clinical information that accompanied the specimens was the level of progression (primary tumors versus nodal metastatic tumors). Each sample was obtained at the time of resection and either immediately flash-frozen and stored in liquid N_2_ or fixed in buffered formalin.

### RT-PCR analysis of GRM1 expression

Total RNA was extracted from tissue and cell lines using RNeasy Plus Mini Kit (Qiagen, Valencia, CA) and subjected to reverse transcription using High-capacity cDNA Reverse Transcription Kit (Applied Biosystems-Life Technologies, Carlsbad, CA). QRT-PCR was performed using TaqMan universal qPCR master mix (Applied Biosystems/Life Technologies, Carlsbad, CA) and the following primers and probes:

***GRM1***

**Sense** 5′-GCA·CGG·CCT·GCA·AAG·AGA·ATG·AAT-3′

**Antisense** 5′-TCC·ACT·CAA·GAT·AGC·GCA·CAG·       GAA-3′

**Probe** 5′-/56-FAM/TCA·CCT·GCA·AAG·CTT·GTG·     ACT·TGG·GA/3BHQ_1/-3′

***β-actin***

**Sense** 5′-TCA·GCA·AGC·AGG·AGT·ATG·ACG·AG -3′

**Antisense** 5′-ACA·TTG·TGA·ACT·TTG·GGG·GAT G-3′

**Probe** 5′-/56-FAM/ACG·GTG·AAG·GTG·ACA·GCA·     GTG·G/3BHQ_1/-3′

Thermal cycling was performed as follows: 10 min. denaturing step at 95°C, followed by 40 cycles of denaturation consisting of 15 s at 95°C; annealing/extension at 60°C for 60 seconds. Product sizes were 126 bp for *GRM1* and 264 bp for β-actin. Lack of contaminating genomic DNA was verified using no-RT controls. Each sample was run in triplicate and C_t_ determined for *GRM1* and β-actin. The *GRM1*/β-actin ratio was estimated using the ΔΔC_t_ method as described [34], [35].

### Immunoblotting

Protein lysates were collected by scraping cells in RIPA lysis buffer (Santa Cruz, CA). Protein (30 to 100 µg) was separated by SDS-polyacrylamide gel electrophoresis (4 to 20%) and transferred to polyvinylidene fluoride membranes. Immunodetection of mGluR1 (BD Pharmagen, San Jose, CA), and phosphorylated or total ERK (Cell Signaling, Canton, MA), was performed using primary antibodies to these antigens with appropriate secondary antibodies followed by detection using chemiluminescence. Primary blots were stripped and reprobed with antibody against α-tubulin (Sigma-Aldrich, St. Louis, MO) or GAPDH (Novus Biologicals, Littleton, CO).

### Immunohistochemistry and CTMA4 breast tissue microarray

Additional paraffin-embedded breast cancer specimens corresponding to the frozen tissue samples used in the qRT-PCR studies were obtained from the Tissue Retrieval Service. In addition, a breast cancer tissue array (CTMA4) was constructed and contained 580 cores representing approximately 145 cases (4 cores per case), consisting of 115 cases of invasive ductal carcinoma, 15 cases of invasive lobular carcinoma, one case of atypical hyperplasia, 12 cases of ductal carcinoma *in situ* and 2 cases of soft tissue metastasis. The array has been validated, with each spot examined and confirmed to consist of at least 80% cancer cells. 4 µm paraffin sections were cut and mounted on glass slides, deparaffinized, and treated with Protease 1 at 37°C before anti-mGluR1 was applied. Staining was detected using Ventana's prediluted HRP-tagged universal secondary antibody followed by incubation in DAB using the DAB MAP kit (Ventana Medical Systems). A breast cancer pathologist read the array in a blinded fashion.

### Cell proliferation and growth

Cells under the experimental conditions tested (described in the text) were plated at a density of 1×10^4^ cells per well in 96-well tissue culture plates (Corning Incorporated, Corning, NY). Cells were transduced with Lentiviral vectors expressing either GRM1 or control, and the proliferation of the pooled cell populations was determined using the Cell Titer Non-Radioactive Cell Proliferation Assay (MTT Assay) according to manufacturer's protocol (Promega, Madison, WI). In addition, cell growth was measured by plating 5×10^4^ cells in 6-well tissue culture plates (Corning Incorporated, Corning, NY), after which cells were trypsinized and counted every day using a Coulter counter. (Beckman-Coulter, Brea, CA). Similarly, cells were transduced with GIPZ Lentiviral vectors expressing either shRNA against *GRM1* or scrambled control.

### Migration/invasion assays

For migration and invasion assays, 2.5×10^5^ MCF-10 series cells transduced with either pLenti-*GRM1* or, pLenti-LacZ, or sh*GRM1* or control nonsilencing vector were plated on cell inserts with polycarbonate membranes (0.8 µm pore size, BD Biosciences, San Jose, CA).For invasion assays cell inserts were coated with Matrigel (0.8 µm pore size, BD Biosciences, San Jose, CA). Cells were allowed to invade the Matrigel or migrate towards serum (10%) as the chemoattractant for 16 to 24 hours after which non-migrating or non-invading cells were scraped off and the cells on the other side of the membrane were stained by H&E and the migrated and invaded cells were counted manually. For all experiments, to exclude the possibility that the effect of migration or invasion may be because of the differences in cell counting or the functional rate of proliferation, we plated an MTT assay in parallel with migration and invasion assays and confirmed that there were no differences in seeding densities and cell proliferation during incubated times between experimental groups.

### Soft agar assay and live cell imaging

MCF-10 series cells either (1) stably transduced with either *GRM1* or control *LacZ*; (2) stably transduced with sh*GRM1* or control nonsilencing vector; or (3) treated with BAY, were assayed for anchorage-independent growth in a 0.4% agar solution in six well plates. Selection media or BAY-supplemented media was fed thrice a week for three weeks. After three weeks, colonies were stained with *p*-iodonitrotetrazolium violet (Sigma-Aldrich, St. Louis, MO) overnight, and counted on Gel Count machine (Oxford Optronix, Oxford, UK). For 3D live cell cultures of MCF10A series cells, 1.5×10^3^ cells were seeded on 12 mm coverslips precoated with rBM (Cultrex®, R & D Systems, Minneapolis, MN) followed by the addition of a 2% rBM overlay [29]. The morphology of cells and degree of colony formation in the 3D rBM were assessed by visual examination of 16 contiguous fields under differential interference contract (DIC) microscopy at day 4 in culture as described [36]. All images were obtained using a Zeiss LSM-510 META confocal microscope.

### Xenografts

MCF10AT1 cells (1×10^7^), wild-type or stably transduced with either pLenti-*GRM1* or pLenti-*LacZ*, were suspended in 0.5 mL of reconstituted basement membrane (BD Matrigel, BD Biosciences, San Jose, CA) into both flanks of female athymic nude (nu/nu) mice, aged 6 and 8 weeks (Taconic Farms Inc., Germantown, NY). Mice were observed for lesion formation twice a week and euthanized after eight weeks, when lesions were harvested at the injection site, fixed in buffered formalin, and embedded in paraffin for histological examination. Lesions were then examined microscopically for foci of carcinoma.

### Statistics and data analysis

All *in vitro* studies were repeated at least three times and, unless otherwise noted, statistical significance was determined either by one-way ANOVA, chi-square, or unpaired Student's t-test, as appropriate.

## Results, Discussion, and Conclusions

### *mGluR1* expression and activity in breast cancer

**Figure 1:**
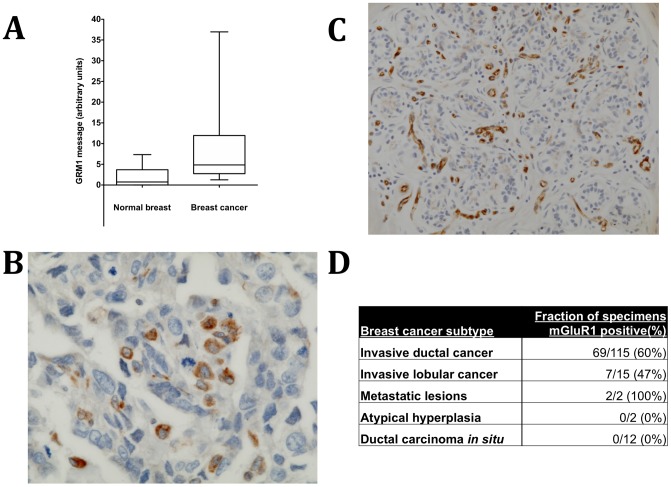
*GRM1* and mGluR1 expression and activity in breast cancer. **A. *GRM1* message is expressed at significantly higher levels in breast cancer versus normal breast.** GRM1 message was measured by qRT-PCR in 49 human breast cancers and 10 normal breast specimens. The whiskers in the box-and-whiskers plot represent a percentile range from 10 to 90. Cancer and control groups were compared using the unpaired t-Mann-Whitney test with Welch's correction (p = 0.0007, arbitrary units normalized to GAPDH ± SEM). **B. mGluR1 expression in human breast cancer.** Staining was patchy, with some breast cancer cells showing intense staining and others showing light staining, suggesting a mosaic of mGluR1-positive and mGluR1-negative cells in this specimen (magnification: 100×). C. mGluR1 expression in normal breast tissue. Little to no staining was observed in normal ductal and lobular epithelial cells. However, there was significant staining observed in the tissue vasculature, primarily the epithelial cells. (Magnification 40×) **D. mGluR1 expression in a breast cancer tissue microarray.** The CTMA4 breast cancer tissue array was subjected to mGluR1 IHC as described in Materials and Methods and the TMA examined by a breast pathologist (N. B.).

To determine whether mGluR1 is expressed in human breast cancer we measured *GRM1* mRNA by qRT-PCR in 49 flash-frozen human breast cancer specimens and 10 specimens of normal breast taken from reduction mammoplasties. Significantly higher *GRM1* message was detected in the invasive cancers (**Figure 1A**). mGluR1 protein expression was also detected by immunohistochemistry (IHC) in primary cancers (**Figure 1B**) and metastatic lesions (not shown). In carcinoma, mGluR1 expression was patchy, with some breast cancer cells showing intense staining and others showing minimal staining, suggesting a mosaic of high-expressing and low-expressing cells. In contrast, in normal breast tissue, virtually no staining could be seen in the actual breast lobules and ducts, although the vasculature of the breast tissue did demonstrate moderate to intense staining with anti-mGluR1 antibody (Figure 1C). IHC performed on the CTMA4 breast cancer tissue array confirmed that mGluR1 expression is common in breast cancer, with 60% of invasive ductal carcinomas and 47% of invasive lobular cancers, but not benign lesions, expressing high levels of mGluR1 (**Figure 1D**).

### mGluR1 expression and activity in a high-expressing TNBC cell line

**Figure 2:**
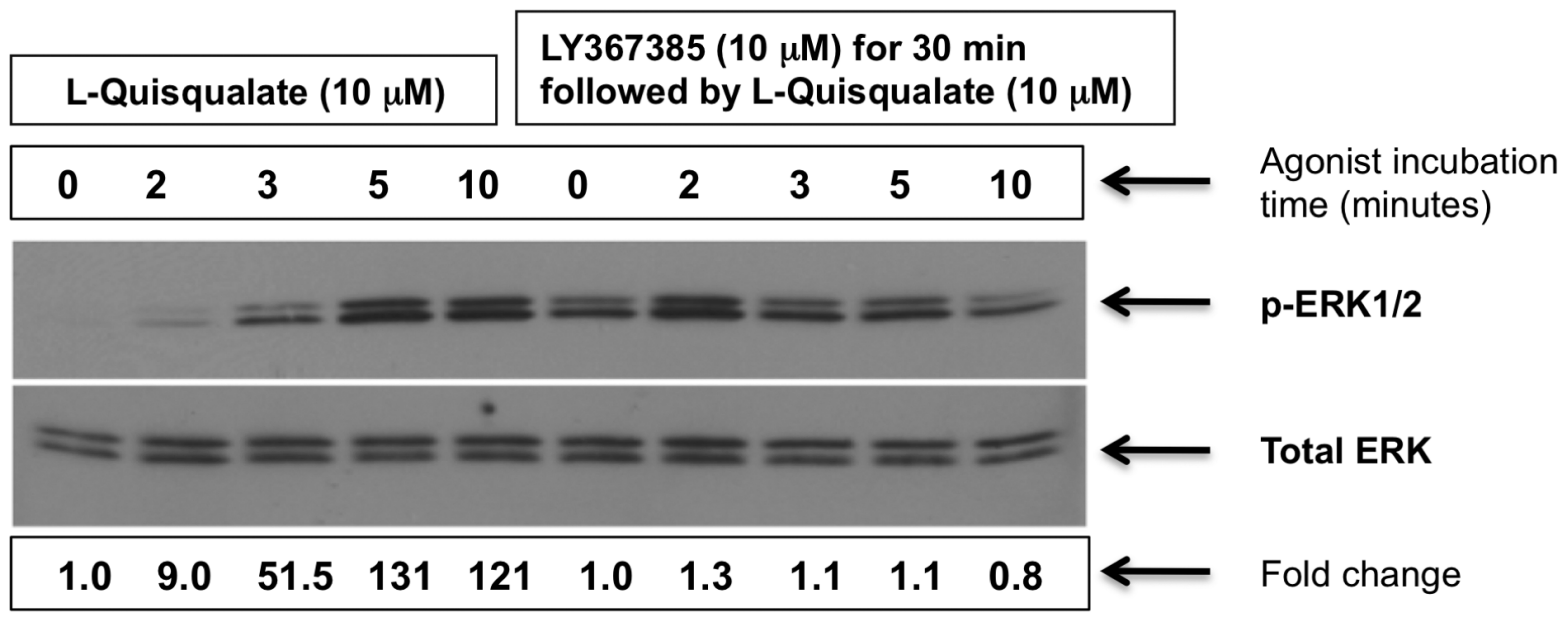
mGluR1 is active in a TNBC cell line. BT549 cells were stimulated in glutamate-free media containing GlutaMAX™ (Life Technologies, Grand Island, NY) with mGluR1 agonist, L-quisqualate (10 µM). Cells were harvested and the fold-increase in phosphorylated ERK1/2 was assayed by Western blot. Pretreatment with mGluR1 antagonist LY367385 for 30 minutes markedly reduced pERK1/2 induction by L-quisqualate (right side of gel). **Bottom gel:** Same blot stripped and re-probed with ERK antibody for normalization. Experiments were repeated two times with similar results.

Next, we tested the effect of modulating mGluR1 signaling in a TNBC cell line (BT549) that was chosen because it endogenously expresses a high level of mGluR1 and is more sensitive to pharmacologic blockade of mGluR1 than other TNBC cell lines we have tested thus far [9]. In addition, BT549 cells grow well in glutamate-free medium supplemented with GlutaMAX™ (Life Technologies Grand Island, NY). BT549 cells were stimulated with the Group I mGluR agonist, L-quisqualate. This resulted in a rapid increase in phosphorylation of mGluR1 downstream target ERK1/2, an effect reduced by pre-incubation with the competitive mGluR1 antagonist LY367385 (**Figure 2**). These results suggest that mGluR1 is active in TNBC.

### mGluR1 expression and activity in an isogenic breast cancer progression series

**Figure 3:**
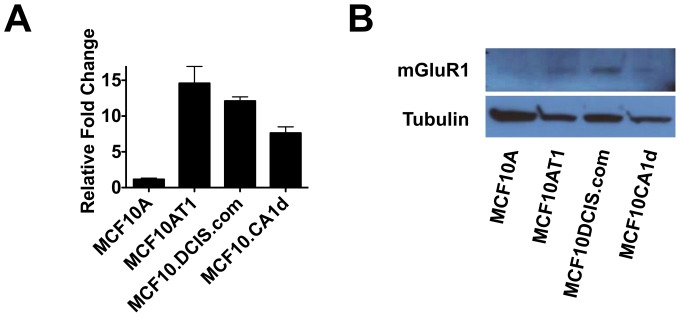
Expression of *GRM1* and mGluR1 in the MCF10 progression series. **A. *GRM1* mRNA expression.** RNA lysates collected from the progression series cells were synthesized to cDNA and qRT-PCR analysis performed as described in Materials and Methods. Relative fold change *GRM1* expression of MCF10AT1, MCF10DCIS.com, and MCF10CA1d are compared to MCF10A cells. **B. mGluR1 protein expression.** Protein lysates were immunoprecipitated with mGluR1 antibody as described in Materials and Methods. Premalignant MCF10AT1 and malignant MCF10DCIS.com, and MCF10CA1d express increased levels of *GRM1* and mGluR1 compared to parental MCF10A cells, with MCF10DCIS.com expressing the highest levels.

To further explore mGluR1 activity in TNBC, in particular, in the progression from normal epithelium to triple negative breast cancer, we measured its expression in the MCF10 progression series of cell lines, which serve as models for mammary epithelial progression [27]. Originally based on observations that human breast cancer cell lines contain mutationally activated ras oncogenes, and many breast carcinomas express higher levels of the ras proto-oncogene protein product than do normal tissues [37], [38], these cell lines were constructed to explore breast cancer progression and include lines representing fibrocystic breast tissue (MCF10A) [24], atypical hyperplasia (MCF10AT1, Ha-*ras/HRAS* transformation of MCF10A) [31], ductal carcinoma *in situ* (MCF10DCIS.com) [39], [40], and invasive TNBC (MCF10CA1d) [33], [41]. Although HRAS is seldom mutated in breast cancer, it is thought that elevated expression could result from upregulated signaling pathways involving these GTPases due to increased coupling to growth factor receptors or other tyrosine kinases commonly overexpressed breast cancer, or increased expression of regulators, the Ras protein itself, or downstream effectors [42], and there is a precedent for cooperation between other genes and HRAS in this series, for example BMI1 [43]. Consistent with a possible role in TNBC progression, *GRM1* mRNA and mGluR1 protein expression was low in MCF10A normal mammary epithelial cells cells compared to premalignant MCF10AT1 cells and malignant MCF10DCIS.com and MCF10CA1d (**Figure 3, A and B**).

**Figure 4:**
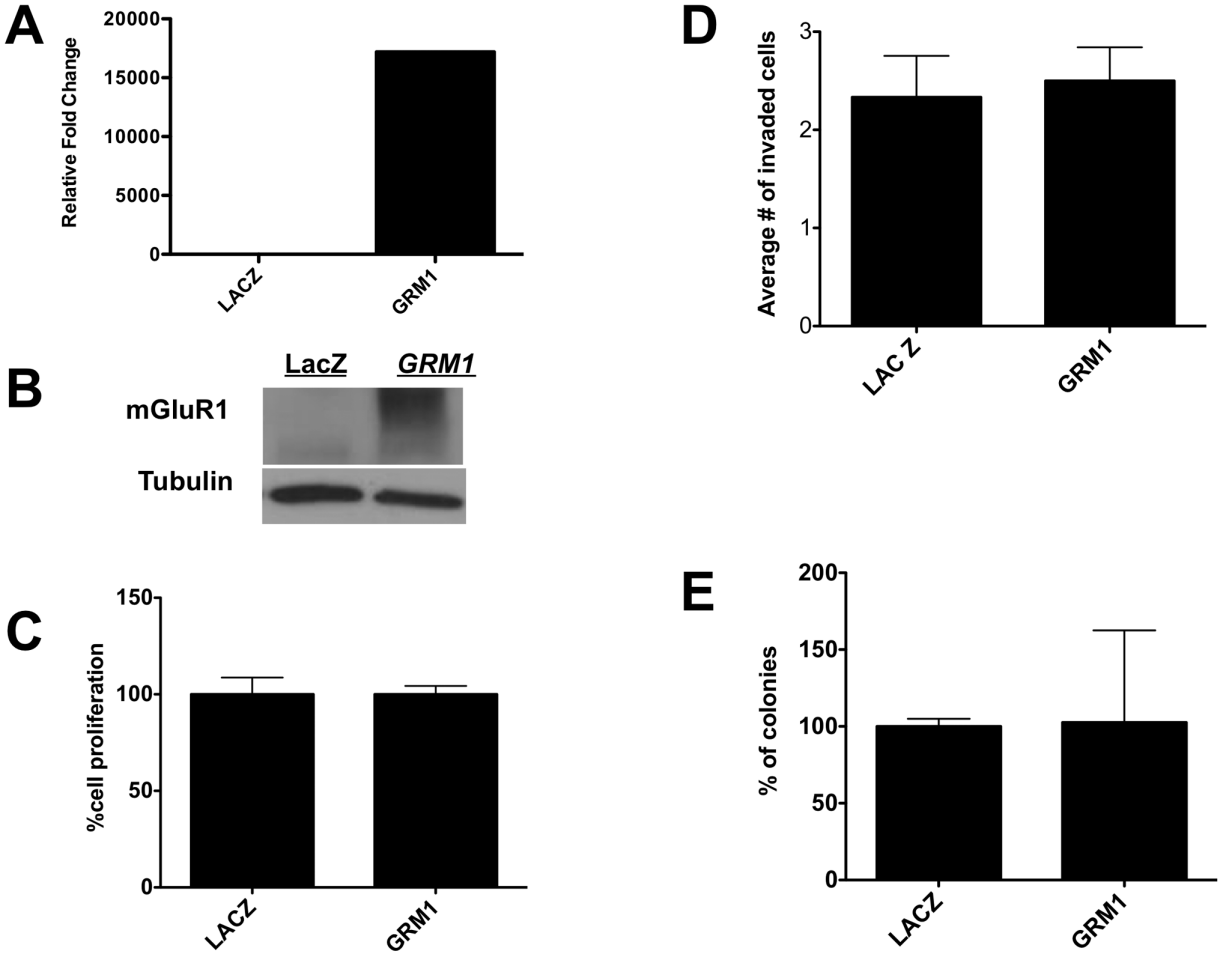
Increasing mGluR1 activity has no effect on measures of oncogenesis in MCF10A cells. **A. *GRM1* expression.** RNA lysates from stable transfectants of *GRM1*-MCF10AT1 or control *LACZ* cells were isolated and subjected to qPCR as described in Materials/Subjects and Methods. *GRM1* is expressed at a level approximately 1.8×10^4^ times higher in GRM1-transduced MCF10A cells than in *LacZ* control infected cells. **B. mGluR1 expression.** Protein lysate (30 µg) was loaded in each lane. The membrane was stripped and reprobed for tubulin antibody to show equal loadings. (***LacZ*** = pLenti-LacZ; ***GRM1*** = pLenti-*GRM1*). **C. Effect of mGluR1 overexpression on proliferation.** Lentivirus-driven mGluR1 expression had no effect on the proliferation of MCF10AT1 cells compared to the control as measured by MTT assay. For MTT assay, 1×10^4^ cells were plated and cell proliferation was analyzed on day 3. **D. Effect of mGluR1 expression on MCF10A migration.** *GRM1* overexpression in MCF10AT1 cells had no effect on MCF10A migration compared to the control *LacZ-*transduced cells. **E. Effect of mGluR1 expression on anchorage-independent colony formation.** MCF10A cells were transduced with either with pLenti-*GRM1* or pLenti-*LacZ* and plated in soft agar as described in Materials and Methods. *GRM1* overexpression had no effect on the number of colonies on soft agar compared to the control *LacZ* cells. All experimenbts were performed in triplicate and results are recorded as the mean ± SEM of three experiments, where *p<0.05 compared to control.

Next, we modulated mGluR1 expression in MCF10 series cell lines using a Lentiviral expression vector (pLenti-*GRM1*) to overexpress mGluR1 in the benign and premalignant members of the MCF10A series (MCF10A and MCF10AT) and a Lentiviral vector expressing shRNA to silence mGluR1 expression in the malignant members of the series (MCF10DCIS.com and MCF10CA1d) (20). We began with MCF10A cells, in which vector-driven *GRM1* and mGluR1 expression was confirmed (**Figure 4, A and B**). We then tested the effect of overexpressing mGluR1 in MCF10A cells and, disappointingly, observed no significant effect on proliferation (**Figure 4C**), invasion through Matrigel-coated polycarbonate membranes (**Figure 4D**), and colony formation in soft agar (**Figure 4E**). Given this finding, we asked whether mGluR1 might function later during TNBC progression, as represented by MCF10AT1 cells, which represent atypical ductal hyperplasia.

**Figure 5:**
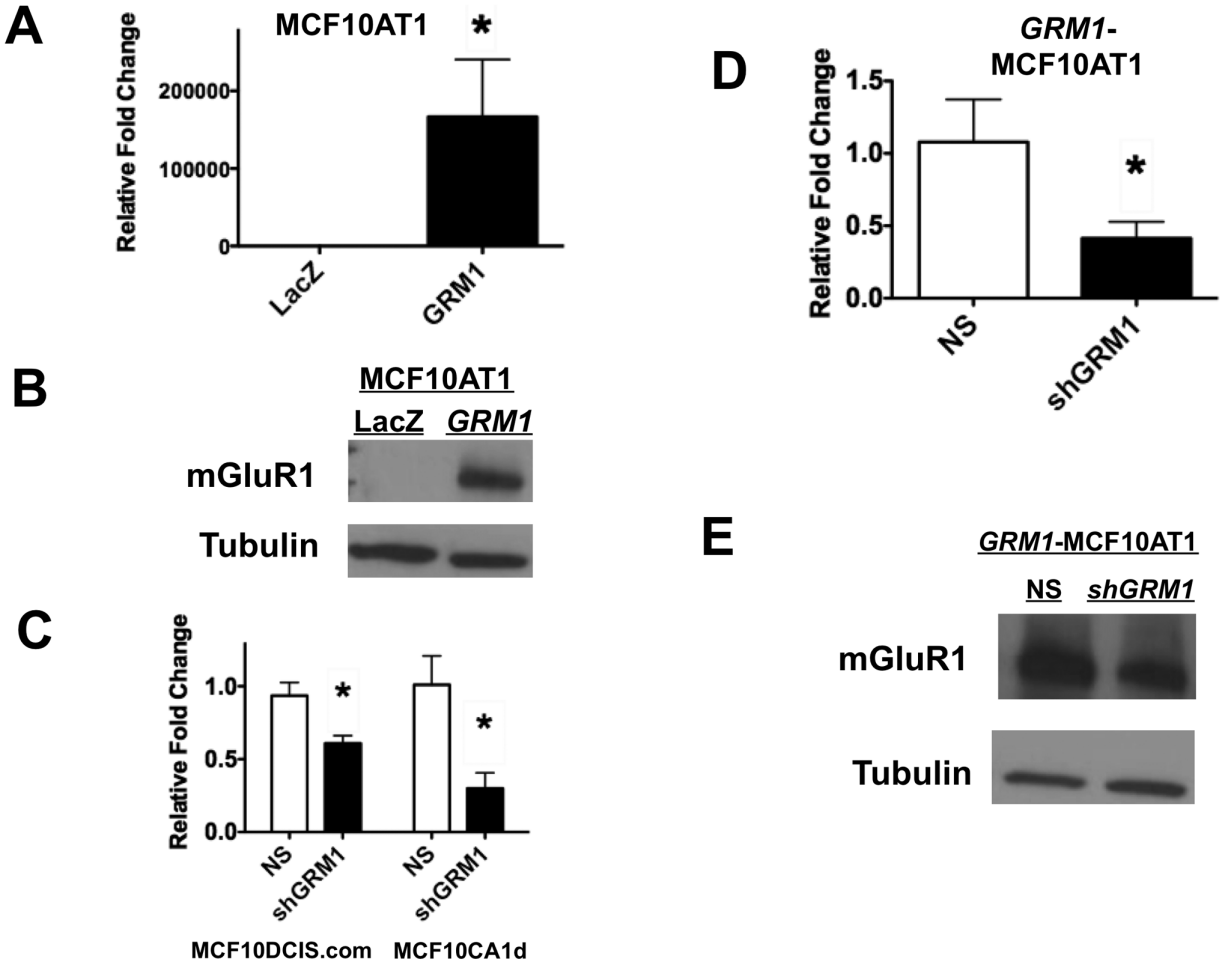
mGluR1 expression and silencing. *GRM1* overexpression results in mGluR1 protein overexpression in MCF10AT1 cells. **A. *GRM1* expression.** RNA lysates from stable transfectants of *GRM1*-MCF10AT1 or control *LACZ* cells were isolated and subjected to QPCR as described in Materials/Subjects and Methods. *GRM1* was expressed at a level 1.6×10^5^ times higher in stable transformed GRM1-MCF10AT1 cells than in *LacZ* control infected cells. **B. mGluR1 expression.** Protein lysate (30 µg) was loaded in each lane. The membrane was stripped and reprobed for tubulin antibody to show equal loadings. (***LacZ*** = pLenti-LacZ; ***GRM1*** = pLenti-*GRM1*). **C, D.** ***GRM1*** **silencing.** RNA lysates from MCF10DCIS.com and MCF10CA1d cells with Lentivirus-driven expression of sh*GRM1* or NS vector were subjected to qPCR analysis. *GRM1* expression was significantly silenced by 0.5 and 0.25 fold in sh*GRM1* transduced MCF10DCIS.com and MCF10CA1d cells respectively, compared to the NS control infected cells (**C**). GRM1-MCF10AT1 cells were silenced with either sh*GRM1* or NS control vector and *GRM1* mRNA message was analyzed using QPCR. *GRM1* is significantly silenced by 0.48 fold in *GRM1-*MCF10AT1 cells compared to the NS control infected cells (**D**). **E. mGluR1 silencing.** Protein lysate (50 µg) was loaded in each lane. mGluR1 is also silenced by sh*GRM1* in *GRM1*-MCF10AT1 cells compared to the NS control cells. qPCR was performed in triplicate and results are recorded as the mean ± SEM of three experiments, where *p<0.05 compared to control. (**For C, D, and E: NS** = pLenti vector containing nonsilencing control: **sh** ***GRM1*** = pLenti vector containing shRNA against *GRM1*.)

**Figure 6:**
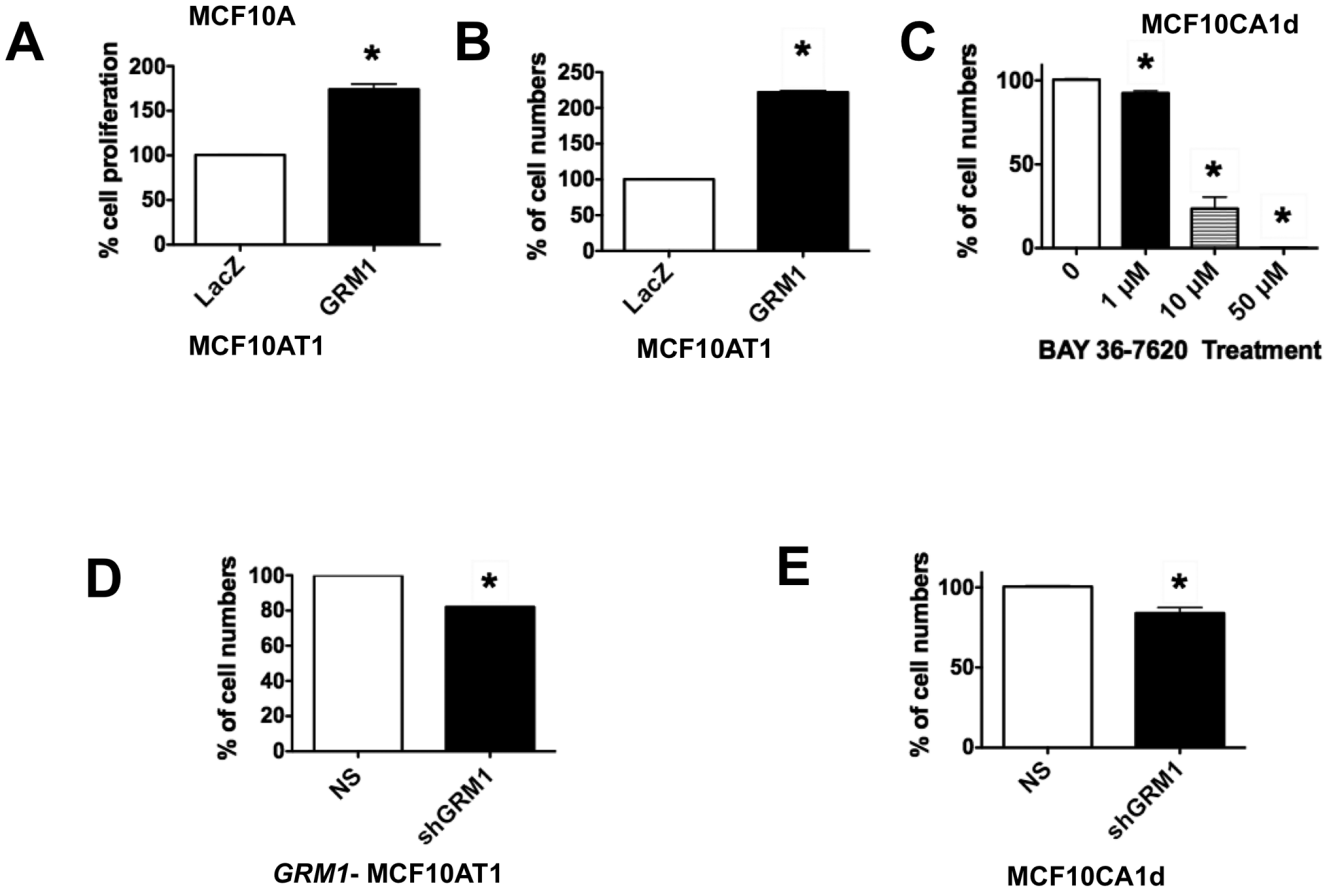
mGluR1 activity regulates cell proliferation. **A. Proliferation.** mGluR1 overexpression significantly increased proliferation of MCF10AT1 cells compared to the control as measured by MTT assay. For MTT assay, 1×10^4^ cells were plated and cell proliferation was analyzed on day 3. **B. Cell counts.** Cell counting by Coulter counter also show that mGluR1 overexpression significantly increased proliferation of MCF10AT1 cells compared to the control LACZ cells. **C. Pharmacological inhibition of mGluR1 inhibits TNBC cell proliferation.** Proliferation, as measured by cell counts of MCF10CA1d cells was significantly inhibited by glutamatergic signaling inhibitor, BAY. **D and E. Effect of silencing mGluR1.** Silencing *GRM1* significantly decreased cell growth compared to the NS vector control in *GRM1-* MCF10AT1 cells (**D**) and in malignant MCF10CA1d cells (**E**). For B, **C, D**, and **E**, growth assays, 5×10^4^ cells were plated in 6-well tissue culture plates and cell numbers counted by Coulter counter on day 3 for B, D, E growth assays and for C growth assay cells were counted on day 6 after plating. Results are recorded as mean ± SEM of replicate experiments, where *p<0.05 compared to control. **Legend**: NS = pLenti vector containing nonsilencing control; sh*GRM1* = pLenti vector containing shRNA against *GRM1*; *LacZ* = pLenti-*LacZ*; *GRM1* = pLenti-*GRM1*.

To address this issue, we studied the effect of modulating mGluR1 expression and activity in MCF10AT1 cells (**Figure 5, A and B**), as well as the ability of the GIPZ shRNA pLenti vector silencing constructs to silence *GRM1* in MCF10DCIS.com and MCF10CA1d cells lines (**Figure 5C**) and in *GRM1* overexpressed MCF10AT1 (*GRM1*-MCF10AT1) cells (see **Figure 5, D and E**). We then transduced MCF10AT1 cells with pLenti-*GRM1* or pLenti-*LacZ*. In contrast to the lack of effect in MCF10A cells compared to the *LacZ* control as measured by MTT assay on day 3 (**Figure 4C**), there was a significant increase in proliferation in *GRM1-*MCF10AT1 cells compared to the *LacZ* control as measured by MTT assay (**Figure 6A**) and cell count (**Figure 6B**) on day 3. Glutamatergic signaling was then inhibited using BAY, resulting in a significant decrease in cell growth over six days compared to controls (**Figure 6C**). To confirm our results, we blocked GRM1 overexpression in the *GRM1*-MCF10AT1 cells using sh*GRM1* and noted a modest but significant decrease in cell number compared to the non-silencing control (**Figure 6D**). A similar decrease in cell growth was also observed in *GRM1*-MCF10CA1d cells silenced with sh*GRM1* compared to the non-silencing vector infected cells (**Figure 6E**). These results suggest that excessive mGluR1 expression and activity play a role in the proliferation of hyperplastic mammary epithelium.

### mGluR1 regulates migration and invasion of MCF10 series cells

**Figure 7:**
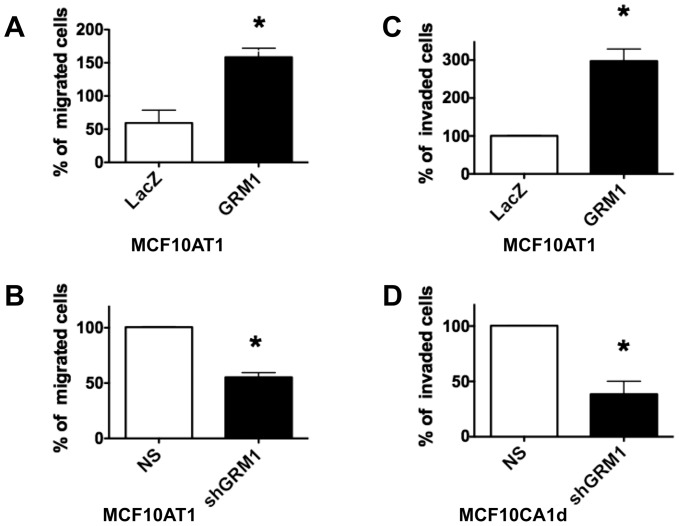
mGluR1 regulates TNBC migration and invasion. MCF10AT1 or MCF10CA1d cells were transduced either with pLenti-*GRM1*, pLenti-*LacZ*, sh*GRM1*, or NS control. 2.5×10^5^ cells were plated on polycarbonate containing cell inserts and migration or invasion towards chemoattractant (10% serum) was measured after 24 hours. For invasion assays, polycarbonate membranes were coated with reconstituted basement membrane. **A. mGluR1 stimulates MCF10AT1 migration.** *GRM1* overexpression in MCF10AT1 cells significantly increased migration of the cells compared to the control *LacZ* cells, which was reversed (**B**) by silencing with sh*GRM1*. **C. MCF10AT1 invasion.** GRM1 overexpression significantly stimulated the invasion of MCF10AT1 cells through polycarbonate membranes coated with reconstituted basement membrane. **D. MCF10CA1d invasion.** *shGRM1* knockdown inhibited the invasion of malignant MCF10CA1d cells compared to the NS transduced cells. Results are recorded as the mean ± SEM of three experiments, where *p<0.05 compared to control. **Legend**: NS = nonsilencing control; sh*GRM1* = shRNA against *GRM1*; *LacZ* = pLenti-*LacZ*; *GRM1* = pLenti-*GRM1*.

Because the effects of inhibiting mGluR1 activity on proliferation were modest, we investigated whether glutamatergic signaling is more important in promoting other oncogenic cell functions. mGluR1 overexpression in MCF10AT1 cells resulted in increased migration (**Figure 7A**), an effect reversed by silencing mGluR1 with sh*GRM1* (**Figure 7B**). Consistent with this result, invasion through reconstituted basement membrane increased when mGluR1 was overexpressed in MCF10AT1 cells (**Figure 7C**), but not in *GRM1*-overexpressing MCF10A (not shown)). Finally, the invasiveness of malignant MCF10CA1d cells decreased when mGluR1 was silenced with sh*GRM1* vector (**Figure 7D**). Our results suggest that mGluR1 activity promotes migration and invasion in premalignant and malignant TNBC cells, but not in nontransformed epithelium.

### Glutamatergic signaling promotes anchorage-independent growth in vitro

**Figure 8:**
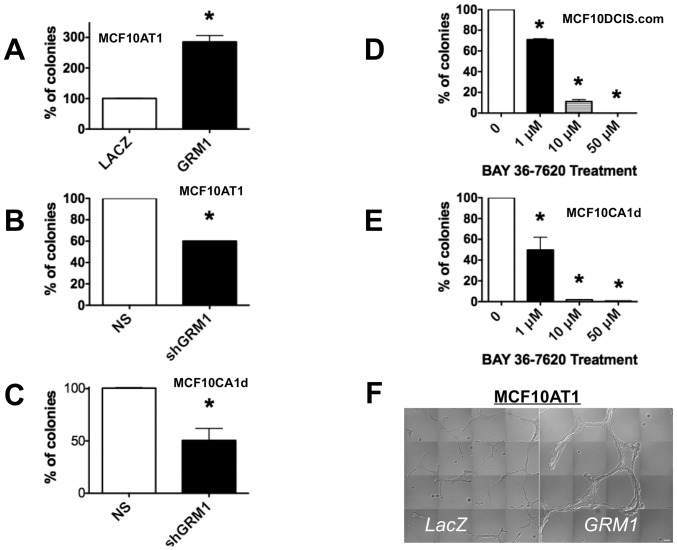
mGluR1 facilitates anchorage-independent growth. **A and B. MCF10AT1 cells.** Cells were transduced with either with pLenti-*GRM1*, pLenti-*LacZ*, sh*GRM1*, or NS control vector and stably selected before plating in soft agar. *GRM1* overexpression significantly increased number of colonies on soft agar compared to the control *LacZ* cells (**A**), an effect reversed by sh*GRM1* (**B**). **C. MCF10CA1d cells.** Silencing *GRM1* significantly decreased colony formation compared to the non-silencing (NS) vector control in MCF10CA1d cells. Finally, colony formation is inhibited by BAY in both MCF10DCIS.com cells (**D**) and MCF10CA1d cells (**E**). For all assays 1×10^5^ cells were suspended in 0.4% agarose and colonies formed on the soft agar were counted after three weeks on Gel Count machine. Results are recorded as the mean ± SEM of three experiments, where *p<0.05 compared to control. **F. Live cell imaging of 4 day MAME cultures of MCF10AT1-** ***LacZ*** **and MCF10AT1-** ***GRM1*** **cells illustrate increases in proliferative and invasive phenotype in cells expressing** ***GRM1*** **.** 15,000 cells were seeded per coverslip on reconstituted basement membrane. Differential Interference Contrast images from 16 contiguous fields were obtained with a Zeiss LSM-510 META confocal microscope. Tiled images allow one to see the relative sizes of the structures formed over a 4-day period. Bar, 100 µm. **Legend**: *LacZ* = pLenti-*LacZ*; *GRM1* = pLenti-*GRM1*.

pLenti-*GRM1* was used to drive mGluR1 expression in MCF10AT1 cells suspended in soft agar and resulted in a 185% increase in colony number compared to control (**Figure 8A**). We then tested the specificity of this effect by silencing overexpressed mGluR1 using sh*GRM1* and observed a 40% decrease in colony formation compared to non-silencing control (**Figure 8B**). Next, we silenced mGluR1 in MCF10CA1d cells and observed a 50% decrease in colony formation (**Figure 8C**). Inhibition of glutamatergic signaling using BAY in MCF10DCIS.com and MCF10CA1d cells also resulted in a dose-dependent decrease in colony formation (**Figure 8, D and E**). Finally, we cultured *GRM1*-transduced MCF10AT1 cells in a 3D reconstituted basement membrane overlay system (3D rBM) [29], [44], [45]. *GRM1*-transduced MCF10AT1 cells formed disorganized, invasive structures with three times the diameter of *LacZ*-transduced controls (**Figure 8F**). Our results suggest that mGluR1 plays a significant role in promoting a more malignant phenotype in cells (MCF10AT1) modeling ADH but not nontransformed mammary epithelial cells (MCF10A)

### mGluR1 contributes to the transformation of hyperplasic MCF10AT1 cells

**Figure 9:**
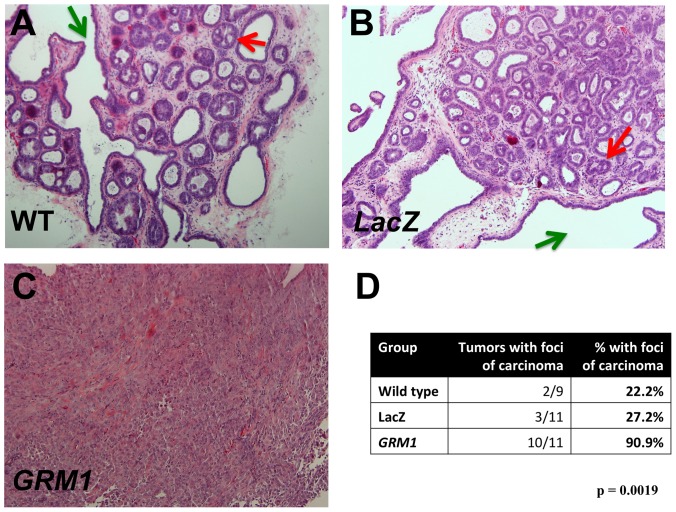
mGluR1 transforms MCF10AT1 cells. MCF10AT1 cells, wild type or transduced with either *LacZ* or *GRM1*, were implanted into both flanks of athymic nude mice and allowed to grow for 8 weeks, after which MCF10AT1 lesions were harvested. **A. Representative wild type MCF10AT1 lesion** with both papillary enfolding (green arrow) and cribiform foci (red arrow). **B.** ***LacZ*** **control MCF10AT1 lesion.** The morphology indicates a hyperplastic lesion with both papillary (green arrow) and initial cribiforming present (red arrow). **C. mGluR1-overexpressing MCF10AT1 lesion.** The morphology indicates invasive cancer. These figures (**A** through **C**) are representative of 10 lesions analyzed for each group (magnification: 100×). **D.** ***GRM1*** **overexpression results in malignant transformation of MCF10AT1 cells.** Standard H&E sections of tumors were examined by a trained observer blinded to experimental group, and MCF10AT1 xenografts assessed for the presence and number of foci of carcinoma.

Finally, we tested whether overexpressing mGluR1 can contribute to the transformation of MCF10AT1 cells. Xenografts from this cell line form glandular structures with varying grades of atypia in nude mice, with approximately 25% of xenografts developing foci of carcinoma over time [28], [31]. MCF10AT1 cells were transduced with pLenti-*GRM1*, and the pooled cell population was implanted into the flanks of athymic nude mice. We chose to use pooled cell populations, rather than picking a series of individual clones, because we were screening for an effect and did not wish to use more animals than necessary to do so. Wild type (**Figure 9A**) and *LacZ*-transduced MCF10AT1 (**Figure 9B**) formed predominantly hyperplastic lesions exhibiting complex patterns of intraductal epithelial proliferations with papillary enfolding and cribiform areas, which is consistent with the original description of xenografts formed by premalignant MCF10AT1 cells [32]. Only 27% and 22% of xenografts derived from LacZ and wild-type controls, respectively, developed foci of invasive carcinoma, rates well within the range of expected results for wild type MCF10AT1 cells and not statistically significantly different from each other (p>0.10, chi-squared). In comparison, xenografts derived from the MCF10AT1 pooled cell population overexpressing mGluR1 developed multiple foci of invasive carcinoma in over 90% of lesions, some of which grew to take over the entire lesion (**Figure 9, C and D**). We note that this result is the more striking in light of our previous report that activated *Ras* and *Neu* showed an additive effect in transformation of MCF10A cells by *in vitro* criteria xenografts but *Ras*- and *Neu*-transduced MCF10AT1 cells were not tumorigenic in nude mice [46]. We have also observed that MCF10AT1 transfected with c-erb2 formed hyperplastic lesions in xenografts with no augmented ability to progress to carcinoma (Miller, unpublished). Our results suggest that mGluR1 can contribute to the transformation of hyperplasic breast epithelium and that its expression contributes to the progression of TNBC.

### Conclusions

Glutamatergic signaling is now known to be important as a regulator of phenotype and proliferation of cells outside the CNS [15]–[18], [47]. Here we report that mGluR1 can also transform hyperplastic mammary epithelium. Our results are particularly relevant to TNBC, for which the identification of a new molecular target would represent a major advance. When mGluR1 was expressed in the parental MCF10A cell line, we did not observe increased proliferation, migration, invasion, or anchorage-independent growth. In marked contrast, when mGluR1 was ectopically expressed in c-Ha-*ras*-transformed MCF10AT1 cells we observed characteristics of a malignant phenotype. These results suggest that, although mGluR1 is clearly oncogenic in melanoma [48], in breast cancer its function appears to be more complex. Consistent with the concept that multiple genetic changes are usually required to transform a normal cell to a malignant phenotype, mGluR1 appears to function in the background of genetic changes that “prime” the cell (in MCF10AT1, c-Ha-*ras*) to respond to the activity of mGluR1 by transformation and there appears to be a dose requirement, given that MCF10AT1 cells already express more mGluR1 than MCF10A. How mGluR1 and c-Ha-*ras* cooperate to result in malignant transformation in TNBC remains to be elucidated.

Our results also have implications for therapy. Currently, unlike the case for ER(+) or HER2(+) breast cancer, there are no validated molecular targets in TNBC, leaving cytotoxic chemotherapy, with all its attendant toxicity, as the only effective systemic therapy. Our results build on our previous results [9] demonstrating that blocking glutamatergic signaling has significant antitumor effects against TNBC by asking converse question, namely what happens when mGluR1 expression increases in nontransformed and premalignant mammary epithelium. We recently reported that riluzole (Rilutek®), an FDA-approved inhibitor of glutamatergic signaling with an extremely favorable safety and side effect profile currently used to treat patients with ALS [49], [50], can inhibit the growth of TNBC xenografts in mice [9]. One potential problem in translating our results to the clinic is that, although BAY is a specific mGluR1 inhibitor, riluzole, the only current inhibitor of glutamatergic signaling in clinical use, is not. This makes comparisons between antitumor effects due to Riluzole or BAY difficult, as riluzole can also inhibit sodium channels [51] and calcium channels [52] as well as PKC activity. It is possible, however, that the less specific inhibitor could end up being the more effective treatment, particularly given its excellent safety and toxicity profile.

Our current results constitute an important observation that argues for a significant role for mGluR1 in TNBC progression and extends our recent results [9] by beginning to elucidate at what point in breast cancer progression mGluR1 functions. We envision repurposing an inhibitor of glutamatergic like riluzole as a treatment for TNBC that is oral, safe, and relatively nontoxic or developing a more highly specific inhibitor of mGluR1, such as BAY, that serves a similar purpose. It might also be possible to combine targeted agents directed at mGluR1 and c-Ha-*ras* signaling in order to produce synergistic toxicity in mGluR1-expressing TNBC.

To our knowledge, this is the first report implicating mGluR1 as a potential oncogene and therapeutic target in breast cancer. It will be important going forward to identify the subtypes of breast cancer that express the most mGluR1 in order to determine the best clinical situations in which inhibition of glutamatergic signaling will have maximal effects and whether somatic mutations in this gene contribute to the development of TNBC, given that such mutations exist in other cancers and TNBC [22], [53]. Future efforts will also focus on isolating individual mGluR1-overexpressing clones and using them to elucidate how mGluR1 interacts with other oncogenes, such as c-Ha-*ras* to transform basal epithelium and how to target glutamatergic signaling, either alone or in concert with other targeted therapies, more effectively in the treatment of breast cancer.
